# Development and Validation of Prediction Models for Severe Obstructive Sleep Apnea Based on Periodic Health Examinations

**DOI:** 10.1111/crj.70177

**Published:** 2026-03-05

**Authors:** Kyoka Kanno, Hiromasa Ogawa, Toshiya Irokawa, Shinya Ohkouchi, Masao Tabata, Natsuko Ohko, Hajime Kurosawa

**Affiliations:** ^1^ Department of Occupational Health Tohoku University Graduate School of Medicine Sendai Japan

**Keywords:** logistic models, mass screening, obstruction, occupational health, polysomnography, predictive value of tests, sleep apnea

## Abstract

**Introduction:**

Obstructive sleep apnea (OSA) is not only associated with reduced work efficiency and an elevated risk of occupational accidents but also with hypertension, diabetes, and other lifestyle‐related diseases, making it an important occupational health concern. Conventional questionnaire–based screening may fail to detect OSA because it frequently lacks subjective symptoms. Herein, we aimed to develop and validate a simple objective, questionnaire‐independent prediction model for severe OSA using periodic health examination (PHE) data.

**Methods:**

Following the Transparent Reporting of a multivariable prediction model for Individual Prognosis Or Diagnosis (TRIPOD), we analyzed the data of 671 patients who underwent overnight polysomnography (PSG) at Tohoku University Hospital. Eight predictors—age group, sex, obesity, hypertension, diabetes mellitus, dyslipidemia, polycythemia, and liver dysfunction—derived from routine PHE items—were included in logistic regression models to predict severe OSA, defined as an apnea–hypopnea index (AHI) ≥ 30 or a 3% oxygen desaturation index (ODI) ≥ 30. Internal validity was assessed using bootstrap samples. External validation was performed using overnight percutaneous oxygen saturation data of 100 university employees.

**Results:**

The areas under the receiver operating characteristic curve were 0.67 and 0.72 for the AHI‐ and ODI‐based models, respectively. The internal validity was generally acceptable. In external validation, the AHI model had a sensitivity and specificity of 1.00 and 0.95, respectively, while the ODI model exhibited values of 0.50 and 0.97, respectively.

**Conclusion:**

We developed and validated two predictive models for severe OSA using the PHE data. These models could be used for screening by occupational physicians and clinicians.

## Graphical abstract

This study developed and validated two prediction models for severe obstructive sleep apnea using data from periodic health examinations. These models enable early detection of high‐risk individuals and may facilitate timely referral and intervention in occupational and clinical settings.

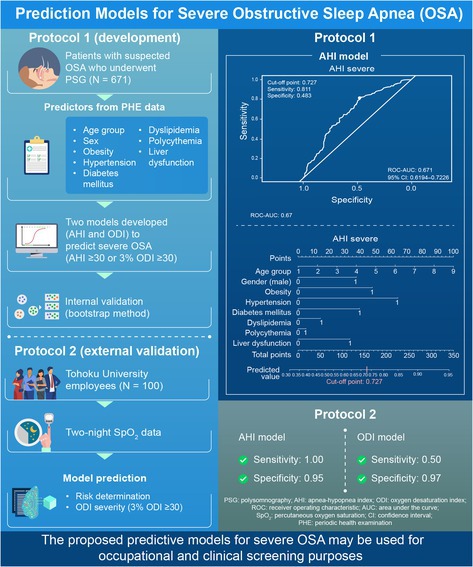

## 1 Introduction

Obstructive sleep apnea (OSA) is characterized by repeated episodes of apnea and hypopnea during sleep due to upper airway obstruction. It is estimated that OSA affects 900 million to 1 billion individuals worldwide [1]. Aging, male sex, and obesity are the major risk factors, and their prevalence is particularly high in middle‐aged and older males.

OSA is reportedly involved in the development and progression of numerous chronic diseases, including hypertension, atrial fibrillation, heart failure, coronary artery disease, diabetes, cognitive dysfunction, and glaucoma. Furthermore, OSA is associated with a substantial socioeconomic impact, given that it increases medical costs, decreases labor productivity, and increases the risk of traffic accidents. Given the importance of early detection and intervention, the development of inexpensive and simple screening methods is required.

It is estimated that there are approximately 9 million individuals with moderate or severe OSA in Japan [1], with only ~500 000 receiving CPAP [2], leaving many untreated potential patients. This is because many individuals with OSA remain unaware of their subjective symptoms, such as sleepiness, despite having the disease.

Subjective screening tests, such as the Berlin Questionnaire (BQ), STOP‐BANG Questionnaire (SBQ), STOP Questionnaire, and Epworth Sleepiness Scale (ESS), are widely used to screen OSA. However, these questionnaires are insufficient for identifying patients with OSA who have few or no subjective symptoms. Objective screening methods for OSA include type 1–4 portable monitoring devices, including the measurement of all‐night percutaneous oxygen saturation (SpO_2_) [3]; however, their widespread application to all workers is limited by costs and the complexity of measurement. Therefore, there is a need to develop a simple, inexpensive, and objective screening tool capable of identifying patients with OSA with few or no subjective symptoms.

Even in the absence of subjective symptoms, severe OSA is associated with hypertension, cardiovascular disease, arrhythmias, impaired cognitive performance, and an increased risk of occupational accidents. Therefore, identifying high‐risk individuals has clinical and public health value, particularly in occupational health systems where early referral, health guidance, and follow‐up can be provided.

In Japan, the health examination system, based on the Occupational Health and Safety Law, and follow‐up measures by occupational physicians play a crucial role in maintaining workers' health and ensuring health and safety in the workplace. Under this law, employers are required to provide employees with periodic health examination (PHE) annually, including physical measurements, blood pressure (BP) measurements, urinalysis, blood tests, electrocardiograms, and chest radiographs. Additionally, business establishments with 50 or more employees are required to appoint an occupational physician. Based on the results of the health examination, industrial physicians will take employment measures such as reducing working hours or transferring work, provide health guidance to improve lifestyle habits, and encourage employees to visit a medical institution.

Assessments performed during PHEs include age, obesity, hypertension, polycythemia, diabetes mellitus, dyslipidemia, and liver dysfunction, which are highly associated with OSA and may facilitate the generation of a predictive model for OSA. Nevertheless, no model capable of predicting OSA based on the PHE results has been established.

In this study, we aimed to develop a simple model to predict severe OSA based on the results of PHEs and to establish an objective screening test that can be administered to all workers. This model may be useful in identifying severe OSA in individuals with few subjective symptoms, particularly by occupational physicians for post‐measures and by general clinicians for screening.

Importantly, the predictors used in the present study are not limited to clinically diagnosed comorbid diseases. Rather, they consist of routine PHE items. These parameters are objectively measured and may reflect subclinical abnormalities even before a disease is formally diagnosed.

## 2 Materials and Methods

This study was conducted according to the Transparent Reporting of a multivariable prediction model for Individual Prognosis Or Diagnosis (TRIPOD).

This study consisted of Protocol 1 (development of the prediction model) and Protocol 2 (validation of the prediction model). Protocols 1 and 2 were approved by the Ethics Committee of the Tohoku University Graduate School of Medicine (2024‐1‐009). Protocol 1 was a retrospective study and, therefore, was addressed based on an opt‐out method, whereby the study content was disclosed on the Tohoku University Graduate School of Medicine Ethics Committee website (https://www.med.tohoku.ac.jp/public/rinri/department/all2024/). Protocol 2 was handled based on an opt‐in method in which all participants were informed orally and in writing regarding the study content, and informed consent was obtained. Protocol 2 was registered in the UMIN Clinical Trials Registry (UMIN‐CTR) (UMIN00051186).

### 2.1 Protocol 1: Model Development and Internal Validation

#### 2.1.1 Study Design

This was an observational (retrospective), exploratory, single‐site study.

#### 2.1.2 Subjects

The subjects were patients with suspected OSA who underwent PSG at Tohoku University Hospital between August 2021 and August 2023. The exclusion criteria were as follows: (1) age < 15 years, (2) hematologic disease, (3) chronic renal failure, and (4) rare disease. Criteria (1) and (4) were set to create a predictive model in the general working population, and Criteria (2) and (3) were established to exclude diseases affecting the “polycythemia” item in the data to be collected. Cases with missing values were excluded from the analysis, and only cases with complete data (complete cases) were used.

#### 2.1.3 Data Collection

Medical history and medications, age, sex, body mass index (BMI), BP, red blood cell (RBC) count, hemoglobin (Hb), aspartate aminotransferase (AST), alanine aminotransferase (ALT), gamma‐glutamyl transpeptidase (γ‐GTP), triglyceride (TG), high‐density lipoprotein‐cholesterol (HDL‐C), low‐density lipoprotein‐cholesterol (LDL‐C), fasting blood glucose (FBG), and hemoglobin A1c (HbA1c) were collected. These are included in the PHE items and are known to be highly associated with OSA. The apnea–hypopnea index (AHI) and 3% oxygen desaturation index (ODI) values were obtained from the PSG results. For reference ranges of each test item, we used the value of “A: Normal” in the “Criteria category (Revised on April 1, 2024)” (https://www.ningen‐dock.jp/en_other_inspection/) of the Japan Society of Ningen Dock and Preventive Medical Care (Table 1). However, given that “RBC” was deleted from the items in 2018, the reference values of “Criteria category (Revised on April 1, 2017)” (https://www.ningen‐dock.jp/ningendock/pdf/973af37ac356d09292413b7f54704df6.pdf) were used. The reference range for BP was adjusted to account for the high prevalence of hypertension, with systolic BP (SBP) of ≤ 139 mmHg and diastolic BP (DBP) of ≤ 89 mmHg as the reference range.

**TABLE 1:** Reference values in this study.

	Males	Females
BMI (kg/m^2^)	≦ 24.9	≦ 24.9
SBP (mmHg)	≦ 139	≦ 139
DBP (mmHg)	≦ 89	≦ 89
RBC (10^6^/μL)	≦ 539	≦ 489
Hb (g/dL)	≦ 16.3	≦ 14.5
AST (U/L)	≦ 30	≦ 30
ALT (U/L)	≦ 30	≦ 30
γ‐GTP (U/L)	≦ 50	≦ 50
TG (mg/dL)	≦ 149	≦ 149
HDL‐C (mg/dL)	≧ 40	≧ 40
LDL‐C (mg/dL)	≦ 119	≦ 119
FBG (mg/dL)	≦ 99	≦ 99
HbA1c (%)	≦ 5.5	≦ 5.5

Abbreviations: γ‐GTP, gamma‐glutamyl transpeptidase; ALT, alanine aminotransferase; AST, aspartate aminotransferase; BMI, body mass index; DBP, diastolic blood pressure; FBG, fasting blood glucose; Hb, hemoglobin; HbA1c, hemoglobin A1c; HDL‐C, high‐density lipoprotein‐cholesterol; LDL‐C, low‐density lipoprotein‐cholesterol; RBC, red blood cell; SBP, systolic blood pressure; TG, triglycerides.

Each variable was categorized to establish a simple predictive model and to account for potential changes in values related to therapeutic interventions.

Age was categorized into 10‐year age groups as follows: 15–19, 20–29, 30–39, 40–49, 50–59, 60–69, 70–79, 80–89, and 90–99 years. Sex was coded as 1 for male and 0 for female.

Obesity was defined as a BMI ≧ 25 kg/m^2^. Hypertension was defined as SBP or DBP exceeding the standard values at the initial examination or current treatment for hypertension. Diabetes mellitus was defined as HbA1c exceeding the standard value or current treatment for diabetes mellitus. Dyslipidemia was defined as LDL‐C exceeding the standard value or current treatment for dyslipidemia. Polycythemia was defined as RBC or Hb level exceeding the standard value. Liver dysfunction was defined as AST, ALT, or γ‐GTP exceeding the standard value. The thresholds used for these definitions were based on the reference ranges listed in Table 1. Each variable was coded as 1 (yes) or 0 (no).

OSA severity was classified according to the AHI as follows: mild (5 ≦ AHI < 15), moderate (15 ≦ AHI < 30), and severe (AHI ≧ 30). In this study, severity classification based on the 3% ODI was performed using the same cutoff values as AHI.

#### 2.1.4 Model Development and Internal Validation

Univariate and multivariate logistic regression analyses were performed using AHI‐severe or ODI‐severe as the objective variables, and age group, sex, obesity, hypertension, glucose metabolic disorders, dyslipidemia, polycythemia, and liver dysfunction as explanatory variables to create two models to predict AHI and ODI severity. To determine the accuracy of the prediction models, receiver operating characteristic (ROC) analysis was performed, and the area under the curve (AUC) values were calculated. We used a stepwise method (in both directions) to select variables. The ROC–AUC values were calculated for the model with variable selection using the stepwise method and the model that included all candidate variables, and their predictive performances were compared. Consequently, the model that demonstrated higher forecasting performance was adopted as the final forecasting model. Pseudo‐*R* ^2^ values (Cragg and Uhler's *R* ^2^) were calculated to evaluate the goodness of fit of the prediction models. The optimal cutoff value for determining the presence or absence of risk was determined. Furthermore, two nomograms were created to easily estimate the risk of AHI and ODI severity.

In addition, to assess the internal validity of the created predictive models, a bootstrap method (1000 iterations) was applied to detect overfitting and examine the stability of the predictive performance to confirm the reliability and practicality of the models.

### 2.2 Protocol 2: External Validation

#### 2.2.1 Study Design

This was an interventional, validation, single‐center study.

#### 2.2.2 Subjects

Subjects were recruited from September 2023 to June 2024 through an on‐campus bulletin board for the faculty and staff at Tohoku University. Sample size was calculated based on the proportion of OSA‐severe cases (AHI ≥ 30) in the predictive model development study (Protocol 1) and the number of explanatory variables to be included in the predictive model. Because logistic regression generally requires at least 10 events per outcome, it was estimated that at least 80 OSA‐severe cases were needed for this study using eight variables. Given that the proportion of severe OSA cases in the target population for Protocol 1 was estimated to be ~80%, the sample size of 100 was estimated for Protocol 2. These estimates were preliminarily reviewed using the R statistical software. Eligibility criteria were defined as the submission of results of a regular physical examination performed within the previous year and were as follows: (1) age < 15 years, (2) hematologic diseases, (3) chronic renal failure, and (4) rare diseases. Criteria (1) and (4) were set to examine the external validity of the prediction model in the general working population; Criteria (2) and (3) were set to exclude diseases affecting the “polycythemia” category from the data to be collected. Cases with missing values were excluded from the analysis, with only cases with complete data (complete cases) employed.

#### 2.2.3 External Validation of predictive models

Considering the cost and participant burden, a simple all‐night SpO_2_ measurement was used instead of PSG; the 3% ODI was used as an alternative to the AHI as an indicator of sleep‐disordered breathing.

A TEIJIN PULSOX‐Me300 pulse oximeter was used for all‐night SpO_2_ measurement (medical device approval number: 225AABZX00066A01) (PMDA public information) (https://www.info.pmda.go.jp/downfiles/md/PDF/290447/290447_225AABZX00066A01_A_01_03.pdf). A respiratory event was one in which SpO_2_ fell by ≥ 3% from the baseline (3% ODI). The measurements were recorded manually at bedtime and terminated manually upon awakening.

The subjects underwent two nights of all‐night SpO_2_ measurements at home. As the all‐night SpO_2_ measurements did not identify sleep time, the measurement time was corrected based on the bedtime and wake‐up time records. A 3% ODI, which was the higher of the two measurements with fewer measurement errors, was used. PHE results and 3% ODI data were classified as described in Protocol 1. The predictive model of Protocol 1 was used to calculate risk probabilities, and cutoff values were used to determine the presence or absence of risk. The agreement between the results of risk determination by the model and the presence or absence of ODI severity based on the 3% ODI value measured using all‐night SpO_2_ measurements was evaluated. Sensitivity, specificity, positive predictive value (PPV), and negative predictive value (NPV) were calculated. Because AHI cannot be evaluated using all‐night SpO_2_ measurements, the results of the 3% ODI determination were used as a surrogate for AHI to validate the AHI prediction model.

In Protocol 1, the 3% ODI was derived from PSG. In Protocol 2, all‐night SpO_2_ was measured using a portable pulse oximeter (TEIJIN PULSOX‐Me300), and the 3% ODI was calculated based on this device. Although the PULSOX‐Me300 is capable of measuring both 3% and 4% ODI, the 3% criterion, which is the standard metric recommended by the American Academy of Sleep Medicine (AASM), was used to ensure consistency with Protocol 1 and with prior Japanese clinical studies. Direct comparison between PSG‐derived SpO_2_ and PULSOX‐Me300 measurements was not performed.

### 2.3 Statistical Analysis

Clinical characteristics of subjects are expressed as median and interquartile range for continuous variables and number and percentage (%) for nominal variables. For between‐group comparisons, Mann–Whitney's *U* test was used for continuous variables, while Pearson's chi‐square test or Fisher's exact test was used for nominal variables. Data analyses were performed using the JMP Pro 17 software.

In Protocol 1, univariate and multivariate logistic regression analyses were performed to generate prediction equations and nomograms. Stepwise methods (both methods) were used for variable selection. The discriminant performance of the model was evaluated by ROC analysis, and optimal cutoff values were calculated based on the Youden index. For internal validation, the bootstrap method (1000 iterations) was employed to evaluate the presence of overfitting and the stability of prediction performance. Calibration plots were generated to verify the consistency between the predicted and actual incidence rates. These analyses were performed using the R statistical software (version 4.5.1).

The external validation of Protocol 2 evaluated the agreement between the results of risk determination by the model and the presence or absence of ODI severity based on the measured 3% ODI data. Sensitivity, specificity, PPV, NPV, positive likelihood ratio, and negative likelihood ratio were calculated manually from the true positive, false positive, true negative, and false‐negative values based on the formula.

## 3 Results

### 3.1 Protocol 1: Model Development and Internal Validation

Of the 817 patients eligible for Protocol 1, 146 were excluded. Twenty‐nine patients met the exclusion criteria, seven of whom were under 15 years of age, two had hematologic diseases, 15 had chronic renal failure, and five had rare diseases. In total, 117 patients had missing data on the items required for analysis. Accordingly, data from 671 patients were analyzed.

The clinical characteristics of these subjects are presented in Table 2. Six patients had normal AHI, 20 patients had mild AHI, 117 patients had moderate AHI, and 528 patients had severe AHI. Regarding 3% ODI, 48 patients had normal ODI, 146 patients had mild ODI, 208 patients had moderate ODI, and 269 patients had severe ODI.

**TABLE 2:** Clinical characteristics of the Protocol 1 participants.

	All	AHI normal‐moderate	AHI severe	*p*	ODI normal‐moderate	ODI severe	*p*
Total, *n* (%)	671 (100)	143 (21.3)	528 (78.7)		402 (59.9)	269 (40.1)	
Normal		6 (0.9)			48 (7.2)		
Mild		20 (3.0)			146 (21.8)		
Moderate		117 (17.4)			208 (31.0)		
Age, years, median (range)	56 (45–65)	54 (40–63)	56 (46–65)	0.026	56 (44–66)	54 (45–65)	0.672
BMI, kg/m^2^, median (range)	26.8 (23.8–31.0)	25.1 (22.2–28.2)	27.2 (24.2–31.4)	< 0.001	25.4 (22.6–28.2)	29.7 (26.0–34.5)	< 0.001
Sex (male), *n* (%)	404 (60.2)	77 (53.9)	327 (61.9)	0.080	227 (56.5)	177 (65.8)	0.016
Obesity, *n* (%)	429 (63.9)	74 (51.8)	355 (67.2)	< 0.001	214 (53.2)	215 (79.9)	< 0.001
Hypertension, *n* (%)	412 (61.4)	64 (44.8)	348 (65.9)	< 0.001	222 (55.2)	190 (70.6)	< 0.001
Diabetes mellitus, *n* (%)	495 (73.8)	90 (62.9)	405 (76.7)	< 0.001	274 (68.1)	221 (82.2)	< 0.001
Dyslipidemia, *n* (%)	397 (59.2)	76 (53.2)	321 (60.8)	0.099	225 (56.0)	172 (63.9)	0.040
Polycythemia, *n* (%)	128 (19.1)	24 (16.8)	104 (19.7)	0.432	66 (16.4)	42 (23.1)	0.032
Liver dysfunction, *n* (%)	290 (43.2)	49 (34.3)	241 (45.6)	0.015	141 (35.1)	149 (55.4)	<0.001

*Note:* Data are expressed as median and interquartile range for continuous variables and as number and frequency (%) for categorical variables. Continuous variables were evaluated using the Mann–Whitney *U* test, and categorical variables were evaluated using Pearson's x2 test.

Abbreviations: AHI, apnea–hypopnea index; BMI, body mass index; ODI, oxygen desaturation index.

Compared with the AHI normal to moderate group, the AHI‐severe group had significantly higher median age and BMI, and higher prevalence rates of obesity, hypertension, diabetes mellitus, and liver dysfunction. Compared with the ODI normal to moderate group, the ODI severe group exhibited a significantly higher prevalence of median BMI, sex (male), obesity, hypertension, diabetes mellitus, dyslipidemia, polycythemia, and liver dysfunction. No significant differences in median age were detected.

Table 3 presents the results of the univariate and multivariate logistic regression analyses with severe AHI or ODI as the objective variables and age, sex (male), obesity, hypertension, diabetes mellitus, dyslipidemia, polycythemia, and liver dysfunction as explanatory variables.

**TABLE 3:** Results of the univariate and multivariate logistic regression analyses.

	AHI severe						ODI severe					
	Univariate analysis			Multivariate analysis			Univariate analysis			Multivariate analysis		
Variable	SPRC	OR	*p*	SPRC	OR	*p*	SPRC	OR	*p*	SPRC	OR	*p*
Age group	0.17	1.2	0.004	0.13	1.1	0.073	0.03	1.0	0.513	0.11	1.1	0.107
Sex (male)	0.31	1.4	0.102	0.39	1.5	0.055	0.38	1.5	0.019	0.40	1.5	0.029
Obesity	0.68	2.0	< 0.001	0.50	1.7	0.016	1.26	3.5	< 0.001	1.13	3.1	< 0.001
Hypertension	0.87	2.4	< 0.001	0.67	2.0	0.001	0.66	1.9	< 0.001	0.50	1.6	0.008
Diabetes mellitus	0.69	2.0	< 0.001	0.42	1.5	0.062	0.79	2.2	< 0.001	0.61	1.9	0.004
Dyslipidemia	0.31	1.4	0.101	0.15	1.2	0.454	0.34	1.4	0.036	0.19	1.2	0.283
Polycythemia	0.21	1.2	0.399	0.05	1.1	0.853	0.43	1.5	0.029	0.12	1.1	0.595
Liver dysfunction	0.49	1.6	0.012	0.35	1.4	0.110	0.84	2.3	< 0.001	0.64	1.9	< 0.001

Abbreviations: AHI, apnea–hypopnea index; ODI, oxygen desaturation index; OR, odds ratio; SPRC, standardized partial regression coefficient.

When the AHI severity was used as the objective variable, univariate logistic regression analysis revealed that all variables had positive standard partial regression coefficients, with significant differences in age, obesity, hypertension, diabetes mellitus, and liver dysfunction (Table 3).

The multivariate logistic regression analysis showed that all variables had positive standard partial regression coefficients, with significant differences in obesity and hypertension (Table 3). The regression equations obtained are as follows:

$$P=\frac{1}{1+e^{-x}}$$

$$\begin{array}{c} x=-0.768+0.132\times \left[\mathrm{Age}\;\text{group}\right]+0.395\times \left[\text{Male}\right]+0.503\times \left[\text{Obesity}\right]\\ \;+0.674\times \left[\text{Hypertension}\right]+0.415\times \left[\text{Diabetes mellitus}\right]\\ \;+0.151\times \left[\text{Dyslipidemia}\right]+0.050\times \left[\text{Polycythemia}\right]\\ \;+0.352\times \left[\text{Liver Dysfunction}\right] \end{array}$$

P=Probability of severeAHI

The pseudo‐*R* ^2^ (Cragg & Uhler) was 0.103.

**FIGURE 1:**
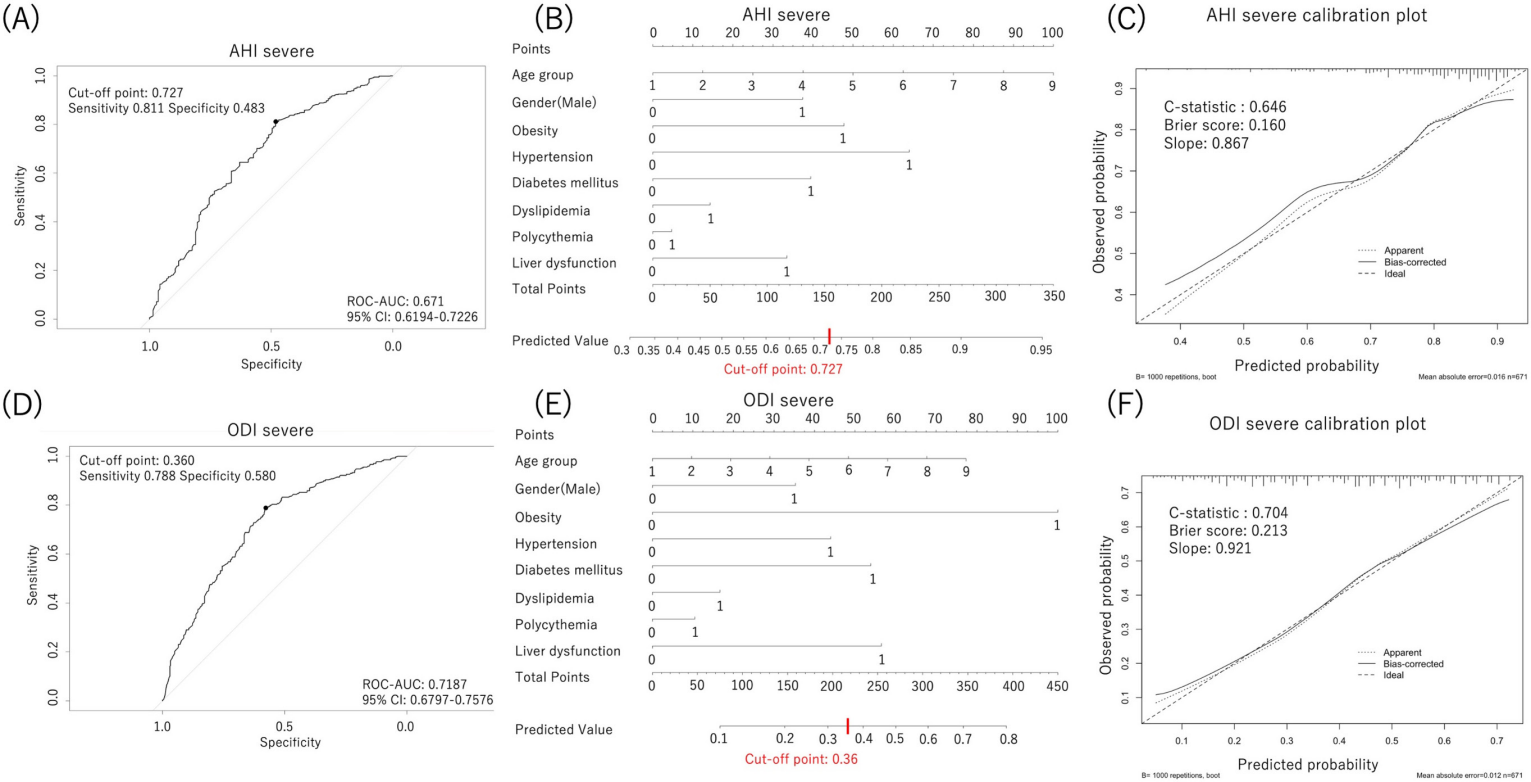
Protocol 1 ROC curves and nomograms. (A) AHI severe discrimination ROC curve. (B) AHI severe discrimination nomogram. (C) Calibration plot for the AHI severe model. (D) ODI severe discrimination ROC curve. (E) ODI severe discrimination nomogram. (F) Calibration plot for the ODI severe model. Instructions for nomogram: Age is categorized by decade as “1, 2, 3, 4, 5, 6, 7, 8, 9” (age group). For each age group, the number corresponding to the subject's age was marked. For other items, mark “1” if applicable and “0” if not. For each item, draw a vertical line from the marked point to the “Points” axis and decide on the number of points. Mark the total points of all items on the “Total points” axis. The predicted value can be obtained by drawing a vertical line from that point to the “Predicted Value” axis.

The ROC curve is shown in Figure 1A, with an AUC of 0.671 and a 95% confidence interval (CI) of 0.619–0.723.

The stepwise model generated the following equation:

$$P=\frac{1}{1+e^{-x}}$$

$$\begin{array}{c} x=-0.698+0.132\times \left[\mathrm{Age}\;\text{group}\right]+0.379\times \left[\text{Male}\right]+0.520\times \left[\text{Obesity}\right]\\ \;+0.684\times \left[\text{Hypertension}\right]+0.429\times \left[\text{Diabetes mellitus}\right]\\ \;+0.375\times \left[\text{Liver Dysfunction}\right] \end{array}$$

P=Probability of severeAHI

Given that the ROC‐AUC was 0.670 and had a lower predictive performance than the all‐variable model, the all‐variable model was used as the final predictive model.

The optimal cutoff value for AHI severity discrimination based on the Youden index was 0.727, with a sensitivity and specificity of 0.811 and 0.483, respectively. A nomogram of this predictive model is shown in Figure 1B.

The *C* statistic for the AHI‐severe model was 0.646, and in the calibration plot (Figure 1C), the predicted and observed probabilities were generally in good agreement. Based on the above observations, the model was judged to have a relatively small effect on overlearning, and its internal validity was judged to be within an acceptable range.

In the univariate analysis of ODI severity, all variables had positive standard partial regression coefficients, with significant differences in sex (male), obesity, hypertension, diabetes mellitus, dyslipidemia, polycythemia, and liver dysfunction (Table 3). The multivariate analysis of ODI severity also demonstrated that all variables had positive standard partial regression coefficients, with significant differences in sex (male), obesity, hypertension, diabetes mellitus, and liver dysfunction (Table 3). The regression equations obtained were as follows:

$$P=\frac{1}{1+e^{-x}}$$

$$\begin{array}{c} x=-3.16+0.109\times \left[\mathrm{Age}\;\text{group}\right]+0.398\times \left[\text{Male}\right]+1.129\times \left[\text{Obesity}\right]\\ \;+0.497\times \left[\text{Hypertension}\right]+0.608\times \left[\text{Diabetes mellitus}\right]\\ \;+0.189\times \left[\text{Dyslipidemia}\right]+0.118\times \left[\text{Polycythemia}\right]\\ \;+0.638\times \left[\text{Liver Dysfunction}\right] \end{array}$$

P=Probability of severeODI

The pseudo‐*R* ^2^ was 0.184. The ROC curve is shown in Figure 1D, with an AUC of 0.719 (95% CI: 0.680–0.758).

The stepwise model for ODI was:

$$P=\frac{1}{1+e^{-x}}$$

$$\begin{array}{c} x=-3.011+0.103\times \left[\mathrm{Age}\;\text{group}\right]+0.369\times \left[\text{Male}\right]+1.147\times \left[\text{Obesity}\right]\\ \;+0.508\times \left[\text{Hypertension}\right]+0.620\times \left[\text{Diabetes mellitus}\right]\\ \;+0.667\times \left[\text{Liver Dysfunction}\right] \end{array}$$

P=Probability of severeODI

As the ROC‐AUC was 0.717 and had a lower predictive performance than the all‐variable model, the all‐variable model was used as the final predictive model.

The optimal cutoff value for severe ODI discrimination based on the Youden index was 0.360, with a sensitivity and specificity of 0.788 and 0.580, respectively. A nomogram of this predictive model is shown in Figure 1E.

A bootstrap method (1000 iterations) was used to assess the internal validity of these models.

The *C* statistic of the ODI‐severe model was 0.704, and the calibration plot (Figure 1F) also demonstrates good agreement between the model predictions and observed values. Accordingly, the model was less affected by overlearning and demonstrated generally good internal validity.

A simplified calculation table of these predictive models is shown in Table 4.

**TABLE 4:** Nomogram simple calculation table.

Category	Items		Value	AHI points	ODI points
Age group	Age (years old)	10–19	1	0	0
		20–29	2	12.5	9
		30–39	3	25	19
		40–49	4	37.5	28
		50–59	5	50	38
		60–69	6	62.5	48
		70–79	7	75	57.5
		80–89	8	87.5	67.5
		90–99	9	100	77
Sex	Sex	Male	1	37.5	35
		Female	0	0	0
Obesity	BMI (kg/m^2^)	> 24.9	1	47.5	100
		≦ 24.9	0	0	0
Hypertension	Treatment	Under treatment	1	64	43
	SBP (mmHg)	> 139			
	DBP (mmHg)	> 89			
	Other than the above		0	0	0
Diabetes mellitus	Treatment	Under treatment	1	40	53
	FBG (mg/dL)	> 99			
	HbA1c (%)	> 5.5			
	Other than the above		0	0	0
Dyslipidemia	Treatment	Under treatment	1	13	17
	TG (mg/dL)	> 149			
	HDL‐C (mg/dL)	< 40			
	LDL‐C (mg/dL)	> 119			
	Other than the above		0	0	0
Polycythemia	RBC (10^6^/μL)	Male > 539 Female > 489	1	4	11
	Hb(g/dL)	Male > 16.3 Female > 14.5			
	Other than the above		0	0	0
Liver dysfunction	AST (U/L)	> 30	1	33	57
	ALT (U/L)	> 30			
	γ‐GTP (U/L)	> 50			
	Other than the above		0	0	0

*Note:* Instructions: Age is categorized by decade as “1, 2, 3, 4, 5, 6, 7, 8, 9” (Age group). For each age group, the number corresponding to the subject's age was marked. For other items, mark “1” if applicable and “0” if not. For each item, draw a vertical line from the marked point to the “Points” axis and determine the number of points. Mark the total points of all items on the “Total points” axis. The predicted value can be obtained by drawing a vertical line from that point to the “Predicted Value” axis.

Abbreviations: γ‐GTP, gamma‐glutamyl transpeptidase; AHI, apnea‐hypopnea index; ALT, alanine aminotransferase; AST, aspartate aminotransferase; BMI, body mass index; DBP, diastolic blood pressure; FBG, fasting blood glucose; Hb, hemoglobin; HbA1c, hemoglobin A1c; HDL‐C, high‐density lipoprotein‐cholesterol; LDL‐C, low‐density lipoprotein‐cholesterol; ODI, oxygen desaturation index; RBC, red blood cell; SBP, systolic blood pressure; TG, triglyceride.

AHI severe:

Total points

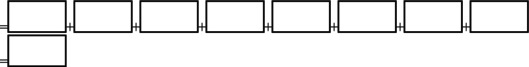

Predicted value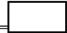 (> 0.727 → AHI severe)

ODI severe:

Total points

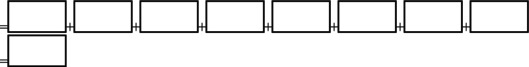
.

Predicted value 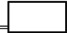 (> 0.360 → ODI severe)

### 3.2 Protocol 2: External Validation

Of the 109 participants, nine were excluded: two met the exclusion criteria (rare disease: two), three were students, and four declined to participate. Finally, 100 participants were included in the validation study.

The clinical characteristics of the patients are presented in Table 5. Based on the 3% ODI, 66 participants had normal ODI, 29 had mild ODI, three had moderate ODI, and two had severe ODI. A markedly lower proportion of participants had severe ODI in Protocol 2 compared with that in Protocol 1. Compared with the ODI normal to moderate group, the ODI‐severe group exhibited significantly higher median BMI, males, and prevalence of obesity, hypertension, and polycythemia. There were no significant differences in the median age and prevalence of diabetes mellitus, dyslipidemia, and liver dysfunction; however, the prevalence of diabetes mellitus and dyslipidemia was higher in the ODI‐severe group.

**TABLE 5:** Clinical characteristics of the Protocol 2 participants.

	All	ODI normal‐moderate	ODI severe	*p*
Total, *n* (%)	100 (100.0)	98 (98.0)	2 (2.0)	
Normal		66 (66.0)		
Mild		29 (29.0)		
Moderate		3 (3.0)		
Age, years, median (range)	48.5 (37.3–54.0)	48.5 (37.8–54.0)	47 (36.0–58.0)	0.749
BMI, kg/m^2^, median (range)	20.9 (19.1–23.4)	20.7 (19.0–23.4)	37.1 (28.7–45.5)	0.020
Gender (male), *n* (%)	16 (16.0)	14 (14.3)	2 (100.0)	0.024
Obesity, *n* (%)	15 (15.0)	13 (13.3)	2 (100.0)	0.021
Hypertension, *n* (%)	10 (10.0)	8 (8.2)	2 (100.0)	0.009
Diabetes mellitus, *n* (%)	36 (36.0)	35 (35.7)	1 (50.0)	1.000
Dyslipidemia, *n* (%)	47 (47.0)	47 (48.0)	0 (0.0)	0.497
Polycythemia, *n* (%)	9 (9.0)	7 (7.1)	2 (100.0)	0.007
Liver dysfunction, *n* (%)	14 (14.0)	13 (13.3)	1 (50.0)	0.262

*Note:* The clinical characteristics of the subjects are expressed as median and interquartile range for continuous variables and number and frequency (%) for categorical variables. Continuous variables were evaluated using the Mann–Whitney *U* test, and categorical variables were evaluated using Fisher's exact test.

Abbreviations: BMI, body mass index; ODI, oxygen desaturation index.

The prediction accuracies of the AHI‐severe and ODI‐severe prediction models compared with the results of all‐night SpO_2_ measurements are shown in Table 6: The sensitivity, specificity, PPV, and NPV of the AHI‐severe prediction model were 1.00, 0.95, 0.29, and 1.00, respectively, while those of the ODI‐severe prediction model were 0.50, 0.97, 0.25, and 0.99.

**TABLE 6:** Efficiency of the AHI severe and ODI severe discrimination model versus the overnight SpO_2_ measurement.

	TP	FN	FP	TN	Sensitivity	Specificity	PPV	NPV	LRP	LRN
AHI severe	2	0	5	93	1.00	0.95	0.29	1.00	19.60	0.00
ODI severe	1	1	3	95	0.50	0.97	0.25	0.99	16.33	0.52

Abbreviations: AHI, apnea–hypopnea index; FN, false negative; FP, false positive; LRP, likelihood ratio positive; LRN, likelihood ratio negative; NPV, negative predictive value; ODI, oxygen desaturation index; PPV, positive predictive value; SpO_2_, percutaneous oxygen saturation; TN, true negative; TP, true positive.

## 4 Discussion

In this study, we developed and validated a simple and easy‐to‐use model for predicting severe OSA based on the PHE results.

The AUCs of both prediction models examined in this study were moderate, suggesting that the discriminative power was limited. Furthermore, the pseudo‐*R* ^2^ values were low, suggesting a limited explanatory power. One contributing factor is that indicators used in similar studies known to be strongly associated with OSA, including neck circumference (NC), subjective symptoms, and nocturnal SpO_2_, were not included in the current study. However, these measures were deemed inappropriate for the purpose of this study because they were procedurally cumbersome, time‐consuming, and were not included in PHE items. Although abdominal circumference is included in PHE, it is not always routinely measured in clinical settings and was not utilized in this study. Nevertheless, the results of the internal validation were generally favorable, and the model was considered to have a reproducible predictive performance for the developed data. Even if the discriminant ability and explanatory power are limited, the model is considered to have a certain level of usefulness as a screening tool if its validity in the field is demonstrated.

External validation revealed that the AHI model had very high predictive accuracy. In contrast, the ODI model indicated a tendency toward a higher false‐negative rate. This may be because the all‐night SpO_2_ measurement used in the external validation was less accurate than PSG and may have been influenced by the variability of cases in the target population. Furthermore, it should be noted that the external validation of this study has limitations owing to the study design, warranting careful interpretation. These limitations are discussed below.

CPAP corrects nocturnal hypoxia and has been shown to improve multiple systemic conditions, including hypertension [4], type 2 diabetes [5], recurrent major cardiovascular and cerebrovascular events [6], atrial fibrillation [7], and glaucoma [8]. Therefore, early screening and intervention are crucial.

In Japan, PHE items are easily administered to numerous subjects to prevent brain, cardiovascular, and lifestyle‐related diseases. Owing to these characteristics, the prediction model established in this study can be operated simply by utilizing existing health examination data and can be introduced into a wide range of settings without requiring special tests or additional costs. Even in regions or populations that lack an adequate health examination system, it enables a simple assessment of the risk of OSA by evaluating items included in the model.

In this study, we used parameters commonly measured in clinical practice during routine physical examinations as predictors. The eight items used were highly associated with OSA [2, 4, 9, 10, 11, 12]. Accordingly, the eight factors in the predictive model are considered appropriate for constructing the predictive model.

Several PHE items previously reported to be associated with OSA, including abdominal circumference, visual acuity, hearing impairment, urinalysis findings, chest radiography abnormalities, and electrocardiographic abnormalities such as atrial fibrillation [7, 8, 13, 14], were not included as predictors in this study because these items were not consistently available for all participants in Protocol 1. Future studies incorporating these routinely collected examination items may further improve the predictive performance of the model.

To date, no study has specifically focused on predicting OSA using PHE items, with most exploring prediction models using clinical data. Table 7 compares the variables used in the prediction models and their prediction accuracies with those of existing studies. Fan et al. [15] created three nomograms to predict OSA using clinical data. The mixed factor nomogram and baseline factor nomogram (BAFN) also comprise predictors obtained from PHE items; BAFN includes age, sex, and BMI, resulting in an AUC of 0.747 for detecting OSA with AHI ≥ 5. However, these methods are used to detect mild‐to‐severe OSA and are likely to detect many non‐treatable OSA cases, which may lead to a tightening of the precision examination and increased healthcare costs. In contrast, we developed a predictive model to detect only severe OSA. Because patients with severe OSA have a particularly high risk of developing cerebrovascular and cardiovascular diseases [25], efficient prevention of cardiovascular events is possible if this predictive model can efficiently identify patients with severe OSA who have no subjective symptoms, leading to diagnosis and treatment. This will lead to improvements in patients' life expectancy, prevention of workforce loss at workplaces, and reduction of medical economic losses; therefore, the predictive model is highly valuable.

**TABLE 7:** Comparison with previous studies.

	This study	Fan et al. [15] mixed factor nomogram	Fan et al. [15] baseline factor nomogram	Fan et al. [15] blood factor nomogram	Song et al. [16]	Liu et al. [17]	Yan et al. [18]	Huang et al. [19]	Ye et al. [20]	Teng et al. [21]	Luo et al. [22]	Xu et al. [23]	Sun et al. [24]
Sample size	671	253	253	253	1769	602	492	480	1647	464	401	2913	1035
Predictor variables (PHE items)	Age group, sex, obesity, hypertension, diabetes mellitus, dyslipidemia, polycythemia, liver dysfunction	Age, sex, BMI, FBG, TC, HDL	Age, sex, BMI	FBG, TC, HDL	Age, BMI, sex	Age, BMI	BMI, hypertension	Age, sex, BMI, RBC	Sex, BMI, hypertension, Hb, TG	BMI, hypertension	Abdominal circumference	Age, sex, BMI, abdominal circumference, blood glucose	Age, sex, diastolic BP, BMI
Predictor variables (non‐PHE items)				Apolipoprotein B	ESS, witnessed apnea, dry mouth, arrhythmia	Total bilirubin, high Berlin score, morning dry mouth, or mouth breathing	Morning dry mouth, nocturnal choking, witnessed apnea, ESS	Mean SpO_2_, % time with SpO_2_ < 90% during sleep, Hct, RDW‐SD	WBC, choking, sleepiness, apnea	ESS, smoking history, dry mouth in the morning, dream recall	Disease duration, smoking history, difficulty falling asleep, fatigue	Neck circumference, insulin, apolipoprotein B	Neck circumference, ESS
Outcome variable	AHI ≧ 30, 3% ODI ≧ 30	AHI ≧ 5	AHI ≧ 5	AHI ≧ 5	AHI ≧ 5	AHI ≧ 5	AHI ≧ 15	AHI ≧ 15	AHI ≧ 30	AHI ≧ 30	AHI ≧ 5, AHI ≧ 15, AHI ≧ 30	AHI ≧ 5, AHI ≧ 15, AHI ≧ 30	AHI ≧ 5, AHI ≧ 15, AHI ≧ 30
AUC	0.671, 0.719	0.750	0.747	0.691	0.7991	0.780	0.976	0.935	0.76	0.820	0.838, 0.799, 0.805	0.84, 0.80, 0.78	0.741, 0.755, 0.778

Abbreviations: AHI, apnea–hypopnea index; BMI, body mass index; ESS, Epworth Sleepiness Scale, FBG, fasting blood glucose; Hb, hemoglobin; Hct, hematocrit; HDL, high‐density lipoprotein; ODI, oxygen desaturation index; PHE, periodic health examination; RBC, red blood cell; RDW‐SD, red cell distribution width–standard deviation; SpO_2_, peripheral capillary oxygen saturation; TG, triglyceride; TC, total cholesterol; WBC, white blood cell.

Although 10 similar studies have predicted OSA using clinical data, these studies included subjective items, time‐constrained and burdensome items for subjects, complex measures, and are not simple or easy‐to‐use tools. Furthermore, the items used in these predictive models, such as NC, ESS, percentage of nighttime SpO_2_ below 90%, mean SpO_2_, coefficient of variation of RBC distribution width, apolipoprotein B, insulin, morning thirst, and dream recall, were not included in the PHE items and could not be directly applied to the PHE results. In comparison, the nomogram in this study is the only predictive model demonstrating some degree of predictive accuracy, while being a simple and efficient tool that uses only items included in PHE and detects only severe OSA.

In clinical practice, the BQ, SBQ, STOP, and ESS are widely used to screen for OSA, with reported sensitivities (specificities) of 84% (38%), 93% (35%), 90% (28%), and 58% (60%), respectively, for detecting severe OSA [26]. However, these questionnaires are subjective, and the target population differs from the objective prediction model developed in this study. The purpose of the prediction model developed in this study was to identify severe OSA without subjective symptoms, and we believe that the developed model complements the existing questionnaire.

Limitations of this study and prospects for future research are as follows:

It is important to establish a screening method for mild OSA from the perspective of preventive and preemptive medicine. Because medical management is required for OSA of moderate or higher severity, it is necessary to develop a predictive model for detecting moderate OSA. However, the patient data collected for the development of the prediction model in this study included a few cases of normal‐to‐mild OSA, complicating the development of a prediction model to detect mild and moderate OSA. Accordingly, although further data are needed to develop models for mild and moderate OSA, the present model remains useful for detecting severe OSA, which is strongly associated with cardiovascular risk and increased mortality [27].

In Protocol 2, considering the cost and participant burden, all‐night SpO_2_ measurement was used instead of PSG, and the 3% ODI was used as a surrogate for AHI as a measure of sleep‐disordered breathing. The 3% ODI obtained from the all‐night SpO_2_ measurements correlated strongly with the AHI obtained from the PSG [28]. However, the 3% ODI obtained from the all‐night SpO_2_ measurements tends to underestimate the AHI from PSG. Although the measurement times were corrected based on sleeping and waking time records, eliminating deviations from the actual sleep times was difficult. Thus, Protocol 2 may have underestimated severe OSA prevalence, and the validity of the AHI severity model could not be fully verified, representing the most notable limitation of this study.

The number of samples needed to confirm the external validity of Protocol 2 was calculated from the proportion of OSA severity in Protocol 1 and the number of items applied to the prediction model. The recruitment of participants for Protocol 2 targeted university faculty and staff and was collected through voluntary applications via an on‐campus bulletin board, which may have included a population that was highly health‐conscious and had more leisure time. The participants were young, had a low male ratio, and had low prevalence rates of obesity, hypertension, diabetes mellitus, dyslipidemia, polycythemia, and liver dysfunction. As a result, Protocol 2 had very few severe cases of OSA. In addition, as mentioned earlier, underestimation of the rate of severe OSA may have been influenced by all‐night SpO_2_ measurements. In summary, the validation results of Protocol 2 should be cautiously interpreted.

Different devices were used to assess oxygen desaturation in the two protocols. In Protocol 1, SpO_2_ was obtained from the PSG system, whereas in Protocol 2, SpO_2_ was measured using the TEIJIN PULSOX‐Me300 portable oximeter. Differences in signal processing and averaging time between these devices may have influenced the detection of short‐duration desaturation events. This may partly explain the discrepancy between the high prevalence of severe OSA defined by AHI and the lower prevalence based on ODI severity. Because direct device comparison was not available in this study, caution is required when interpreting ODI‐based severity across protocols. Nevertheless, the primary aim of this study was to develop a practical screening model using routinely available data, and the observed performance supports its applicability in occupational health settings.

Although a highly accurate PSG evaluation is desirable for developing predictive models, it is unrealistic from the standpoint of cost and examinee burden to conduct PSG widely in an asymptomatic population. Therefore, in Protocol 1, a prediction model was developed for patients who were already suspected of having OSA. Its extrapolation to the general population was limited because of a serious selection bias in the target population.

Accordingly, we tested the external validity of this prediction model in Protocol 2 for the general workforce. Although the results of the external validation of the two prediction models warrant careful interpretation as mentioned above, they both had high specificity and a high level of sensitivity in the AHI‐weighted model, indicating a certain level of usefulness. In the future, it is crucial to further confirm the external validity of the models by undertaking prospective studies on workers with more diverse backgrounds.

## Author Contributions

Kyoka Kanno and Hiromasa Ogawa designed the study and performed the data analysis. Kyoka Kanno collected and curated the data and prepared the original draft. Hiromasa Ogawa supervised the project and secured funding. Toshiya Irokawa, Shinya Ohkouchi, Masao Tabata, Natsuko Ohko, and Hajime Kurosawa contributed to manuscript review and editing. All authors reviewed and approved the final manuscript.

## Funding

This work was supported by Scholarship/research grants.

## Ethics Statement

This study consisted of two protocols: Protocol 1 (development of the predictive model) and Protocol 2 (validation of the predictive model). Both protocols were approved by the Ethics Committee of the Graduate School of Medicine, Tohoku University (Approval No. 2024‐1‐009). Protocol 1 was retrospective. In accordance with the institutional ethical guidelines, the requirement for informed consent was waived, and an opt‐out approach was employed by disclosing information about the study on the website of the Ethics Committee of the Graduate School of Medicine, Tohoku University (https://www.med.tohoku.ac.jp/public/rinri/department/all2024/). Protocol 2 was prospective. All participants received both verbal and written explanations of the study and provided written informed consent prior to enrollment. Protocol 2 is registered in the UMIN Clinical Trials Registry (UMIN‐CTR; UMIN00051186).

## Conflicts of Interest

The authors declare no conflicts of interest.

## Data Availability

Research data are not shared.
